# Postnatal Iron Supplementation with Ferrous Sulfate vs. Ferrous Bis-Glycinate Chelate: Effects on Iron Metabolism, Growth, and Central Nervous System Development in Sprague Dawley Rat Pups

**DOI:** 10.3390/nu13051406

**Published:** 2021-04-22

**Authors:** Shasta McMillen, Bo Lönnerdal

**Affiliations:** Department of Nutrition, University of California, Davis, CA 95616, USA; samcmillen@ucdavis.edu

**Keywords:** iron supplementation, ferrous bis-glycinate, ferrous sulfate, brain, infant nutrition

## Abstract

Iron-fortified formulas and iron drops (both usually ferrous sulfate, FS) prevent early life iron deficiency, but may delay growth and adversely affect neurodevelopment by providing excess iron. We used a rat pup model to investigate iron status, growth, and development outcomes following daily iron supplementation (10 mg iron/kg body weight, representative of iron-fortified formula levels) with FS or an alternative, bioavailable form of iron, ferrous bis-glycinate chelate (FC). On postnatal day (PD) 2, sex-matched rat litters (*n* = 3 litters, 10 pups each) were randomly assigned to receive FS, FC, or vehicle control until PD 14. On PD 15, we evaluated systemic iron regulation and CNS mineral interactions and we interrogated iron loading outcomes in the hippocampus, in search of mechanisms by which iron may influence neurodevelopment. Body iron stores were elevated substantially in iron-supplemented pups. All pups gained weight normally, but brain size on PD 15 was dependent on iron source. This may have been associated with reduced hippocampal oxidative stress but was not associated with CNS mineral interactions, iron regulation, or myelination, as these were unchanged with iron supplementation. Additional studies are warranted to investigate iron form effects on neurodevelopment so that iron recommendations can be optimized for all infants.

## 1. Introduction

Postnatal iron deficiency (ID) adversely affects both physical and cognitive development and should be prevented [1,2,3,4,5]. The American Academy of Pediatrics recommends infants receive iron through liquid supplements or fortified formula to prevent ID [6]. Iron-fortified formulas prevent postnatal ID effectively but provide, on average, 20 times the adequate intake (AI) for infants 0–6 months of age [7,8]. Excess iron can also be harmful to infants, and recent studies report adverse effects of iron supplementation in infants who are not ID [9,10,11,12,13,14]. In response to rising concern regarding the efficacy of blanket iron supplementation in infants, pediatric nutrition researchers as well as expert committees have recommended reevaluation of iron recommendations and stressed the need for postnatal iron supplementation research to identify adverse outcomes and define their biological mechanisms [15,16,17,18,19,20,21,22].

Previous studies on infants have reported reduced growth and deleterious cognitive outcomes due to iron supplementation [9,10,11,12,13,14]. In a randomized controlled trial (RCT), iron-sufficient infants who had received standard iron formula (12.7 mg iron/L) had poorer cognitive outcomes at 10 and 16 years of age [10,11] compared to those who had received low-iron formula (2.3 mg iron/L). Comparable cognitive effects were observed in both rodents and pigs [23,24,25,26]. In these studies, several biological mechanisms may have contributed to the cognitive outcomes, including but not limited to iron under-regulation, iron–mineral interactions, or CNS iron overload-induced oxidative stress.

Iron can compete with other essential trace minerals for absorption and transport and iron loading causes oxidative stress in biological environments through generation of reactive oxygen species (ROS). Systemic and cellular regulators of iron homeostasis work to ensure that the diverse iron needs of all tissues are met and still prevent iron toxicity. In early development, however, iron homeostasis might not be as responsive to elevated body iron stores. Indeed, postnatal iron supplementation increases body iron stores even in iron-replete infants [8,27], and under-regulation of iron metabolism in early life is further supported by postnatal iron regulation studies in humans [28], rats [29,30], mice [31], and piglets [25]. It is possible that under-regulation of iron homeostasis would permit iron overload in the CNS with increased iron intake, and iron uptake in the CNS may also be under-regulated postnatally, as previously indicated in rats [30]. Dysregulation of iron metabolism and iron loading contribute to neurodegeneration by causing oxidative stress [32,33]. Removal of iron from the CNS through iron chelation may even be a promising new therapy for those suffering cognitive effects of neurodegeneration [34]. Studies in rodents have concluded that neonatal iron exposure can promote neurodegenerative disease progression later in life, and this may be a result of neonatal CNS oxidative stress [19,24,26,35,36,37]. In neonatal pigs, increased iron supplementation led to iron loading in the hippocampus, the region that forms memories, as well as markers of lipid peroxidation (a form of oxidative stress), and impaired social behavior after weaning [25]. It was concluded that iron loading in the hippocampus might disrupt cognitive development directly through oxidative stress injury. Cognitive effects of iron supplementation have also been associated with reduced expression of myelin basic protein (MBP) in the hippocampus [38]. In summary, due to under-regulation of iron in early life, postnatal iron supplementation might lead to iron loading, and in the CNS, this could lead to oxidative stress and disrupt myelination, thereby explaining deficits in cognitive development.

The existence of a causal link between early life CNS iron exposure and neurodegenerative disease can only be speculated, but this possibility only highlights the need to study the effects of postnatal iron supplementation [19]. Previous studies in animals have relied on a range of iron intervention designs, but none have closely modeled routine postnatal interventions nor have they accounted for differences in milk iron intake between humans and model species [23,25,35,38,39]. Moreover, the vast majority all studies reporting adverse neurodevelopment effects of iron supplementation in humans have used ferrous sulfate (FS), whereas alternative chemical forms of iron have rarely been explored. Therefore, in addition to FS, we investigated the effects of ferrous bis-glycinate (FC), an amino acid chelated form of iron, which due to its unique absorptive fate may be less likely to cause the adverse effects attributed to FS [40]. FC has been shown to be effective and safe for use in infants as a bioavailable source of iron [41]. Herein, we characterized FS and FC iron supplementation effects on growth, iron status, iron regulation, and neurodevelopment in healthy, nursing rat litters, providing new insight into the activities of exogenous iron during one of the critical windows of development.

## 2. Materials and Methods

### 2.1. Animals

The use of animal models is essential for advancing infant nutrition knowledge because a multitude of ethical and procedural limitations preclude this research in humans. Rats are often preferred for postnatal nutrition research because regular handling of pups is comparatively well-tolerated [42,43]. The use of rats for studying outcomes of postnatal iron supplementation is also reinforced by evidence that mechanisms of iron homeostasis across stages of development are consistent between rats and humans [26,27,28].

Animal procedures for this study were approved by the University of California Davis Institutional Animal Care and Use Committee. Sprague Dawley rats between 8 and 10 weeks of age were obtained from Charles River Laboratories (Wilmington, MA, USA) and maintained on standard 18% protein rodent chow (200 mg Fe/kg diet; 2018, Teklad Diets, Madison, WI, USA) in clear polycarbonate hanging cages at constant temperature (22 °C) and humidity (63%) with standard 12 h light cycles; these conditions applied during habituation, breeding, and throughout the entire postnatal experimental period. Rats were habituated to the vivarium for one week prior to breeding. There were 11 nulliparous female breeders and 9 of them had litters, all of which were used for the experiment. Original litter sizes ranged between 10 to 15 pups. In order to normalize growth between litters, newborn pups born within the same 24 h period were randomly assigned to sex-matched litters of 10 pups. All litters nursed freely throughout the experiment, except for a brief period during daily supplementation. On postnatal day (PD) 2, litters were randomly assigned to supplementation groups (*n* = 3 litters, 10 pups each) to receive 10% sucrose vehicle control (CON) or iron as either ferrous sulfate heptahydrate (Cat#215422-250G, Sigma-Aldrich, St. Louis, MO, USA) or ferrous bis-glycinate chelate (Albion Minerals Ferrochel^®^, Balchem Inc., New Hampton, NY, USA). Littermates were assigned to the same treatment group to avoid coprophagic iron transfer across treatment groups, which would be highly confounding.

Pups were weighed every other day beginning PD 2, and litter average body weight (BW) was used to calculate the supplement volume, which provided 10 mg Fe/kg BW· day. This experimental iron dose for postnatal supplementation was designed to represent the daily iron intake of an exclusively formula-fed infant, after adjusting for known differences in milk iron and iron absorption efficiency between humans and rats. References and calculations for iron dose determination are shown in Table 1 and Equation (1) (below). Iron supplements were prepared in acid-washed glassware by dissolving FS or FC in sterile 10% *w*/*v* sucrose at 6 mg iron/mL. Supplementation was performed by hand-pipetting, at the same time each day from PD 2 through PD 14. To deliver calculated volume, a sterile pipette was placed gently on the roof of the mouth to stimulate natural suckling, and solution was dispensed slowly, allowing swallowing at intervals. On PD 15, pups were fasted for 6 h and euthanized by cardiac venipuncture under deep anesthesia (100 mg ketamine × 10 mg xylazine/kg BW). Hippocampi were dissected immediately from fresh brains and all hippocampi were dissected by the same researcher for consistency.

Rat Pup Supplementation Dose = RM · (IF/HM) = [6.4–14] ≈ 10 mg iron/kg BW(1)

### 2.2. Blood Measurements

Whole blood (*n* = 20 per group) was collected in EDTA tubes (Safe-T-Fill Capillary Blood Collection Systems, RAM Scientific, Nashville, TN), and blood measurements were performed on the day of collection. Hemoglobin was measured by the cyanmethemoglobin method using a commercially available kit (Cat#MAK115-1KT, Sigma-Aldrich, St. Louis, MO, USA). For hematocrit measurement, whole blood (*n* = 20) was collected in heparinized capillary tubes (Fisher Scientific, Pittsburgh, PA, USA), centrifuged, and measured in a hematocrit reader.

### 2.3. Tissue Iron, Zinc, Copper, and Manganese

Tissues (liver, *n* = 12 per group; whole brains, *n* = 12 per group) were flash frozen at time of collection and stored at −20 °C. Sample weights were recorded prior to digestion in HNO_3_ (16 mol/L) at room temperature for 7 d. The HNO_3_ was evaporated at sub-boiling temperatures for 6–8 h [52], and remaining tissue ash was rehydrated with ultrapure water (Milli-Q^®^, Millipore Sigma, Burlington, MA, USA) for quantification of iron, zinc, copper, and manganese by atomic absorption spectrometry (Model Smith-Heifjie 4000, Thermo Jarrell Ash Corporation, Franklin, MA, USA).

### 2.4. Histology

At the time of collection, liver tissue (*n* = 6 per group) and whole brains (*n* = 6 per group) were immersion-fixed in 4% *w*/*v* PFA at 4 °C for 24 h. Tissues were then washed in 1× PBS three times, stored in 70% ethanol at 4 °C, and submitted to the UC Davis School of Veterinary Medicine Anatomic Pathology Laboratory for embedding by standard protocols. Tissue sections were stained for iron by Perls’ Prussian blue method with nuclear fast red counterstain.

### 2.5. Real-Time PCR

Tissue samples (liver, *n* = 7 per group; hippocampus, *n* = 7 per group) were stored in RNAlater^®^ (Sigma-Aldrich, St.Louis, MO, USA) solution at time of collection, kept at 4 °C for 24 h, and then stored at −20 °C until extraction by the TRIzol™ protocol (Invitrogen™, Carlsbad, CA, USA). RNA was reverse transcribed to cDNA using a High-Capacity cDNA Reverse Transcription Kit with RNase Inhibitor (Cat#4374966, Applied Biosystems™, Foster City, CA, USA) as outlined by the manufacturer. RT-PCR reactions were performed using a CFX96 Real-Time PCR System (Cat#1725121, Bio-Rad, Hercules, CA, USA) with iTaq Universal SYBR^®^ Green Supermix to determine relative expression of target transcripts. The fold change in target gene expression was calculated and normalized to *Actb* expression using the 2^∆∆Ct^ method. Primer sequences for target and housekeeping genes are listed in Table 2.

### 2.6. Western Blotting

Tissues (duodenum, *n* = 4 per group; hippocampus, *n* = 6 per group) were flash frozen in liquid nitrogen immediately after collection and stored at −80 °C. Frozen tissue samples were homogenized by bead beating with 5 mm stainless steel beads (Qiagen, Valencia, CA, USA) in Pierce^®^ RIPA Buffer (Cat#PI89900, Thermo Fisher Scientific™, Waltham, MA, USA) with Roche cOmplete™ protease inhibitor cocktail (Cat#NC0969110, Sigma-Aldrich, St. Louis, MO, USA) in a TissueLyser II (Qiagen, Valencia, CA, USA). Following quantification of tissue lysate protein by the Bradford assay, 30 ug protein samples diluted in Laemmli buffer were loaded onto 10% TGX Stain-Free™ polyacrylamide gels (Bio-Rad, Hercules, CA, USA) and separated by electrophoresis under reducing conditions (5% 2-mercaptoethanol). Protein was transferred to nitrocellulose membranes using a Trans-Blot Turbo Transfer System (Bio-Rad). Stain-Free™ blot images were captured using a ChemiDoc MP (Bio-Rad, Hercules, CA, USA) and membranes were blocked with 5% non-fat milk (Sigma-Aldrich, St. Louis, MO, USA) in 0.1% Tween^®^20 PBS (PBST) buffer for 1 h. Blots were washed in PBST and resuspended in primary antibody solution for overnight incubation at 4 °C. Primary antibody solutions were prepared according to the following ratios: rabbit 1:1000 rabbit anti-4-HNE (Cat#ab46545; Abcam, Cambridge, MA, USA), 1:1000 rabbit anti-Slc40a1 (Cat#ab58695; Abcam, Cambridge, MA, USA), and 1:100 mouse anti-Fth1 (Cat#sc-376594; Santa Cruz Biotechnologies, Santa Cruz, CA, USA). Following overnight incubation blots were washed thoroughly with PBST and then treated with horseradish peroxidase-conjugated secondary antibody (1:5000 anti-rabbit or anti-mouse, Sigma-Aldrich, St. Louis, MO, USA) in blocking solution. After a final wash in PBST, SuperSignal™ West Femto Maximum Sensitivity Substrate (Thermo Scientific, Fisher Scientific™, Waltham, MA, USA) was used for chemiluminescent detection of Slc40a1 and 4-HNE protein bands and ECL Plus Reagent (Thermo Scientific, Fisher Scientific™, Waltham, MA, USA) was used for detection of Fth1. Blot images were captured on the ChemiDoc^™^ MP (Bio-Rad, Hercules, CA, USA). Total adjusted band densities of target proteins were analyzed by Image Lab Software (Bio-Rad, Hercules, CA, USA) and normalized to total lane protein using Stain-Free™ blot images [58,59,60].

### 2.7. Protein Carbonyl Content

Protein carbonyl content was quantified in hippocampi (*n* = 6 per group) using an OxiSelect™ Protein Carbonyl ELISA kit (Cat#STA-310; Cell Biolabs, Inc., San Diego, CA, USA) according to the manufacturer’s instructions.

### 2.8. Statistical Analysis

Data were analyzed and plotted in GraphPad Prism (Version 8). A repeated-measures two-way ANOVA with Geisser–Greenhouse correction was used to test for treatment group effect on body weight across the supplementation period. Litters were analyzed as biological replicates, with respective pups as technical replicates when testing for effects on growth. Significant differences in gene and protein expression with treatment were detected with a one-way ANOVA with post hoc Tukey’s test. The Shapiro–Wilk test was used to check for normality, and Kruskal–Wallis tests were used with Dunn’s multiple comparison’s test to detect group differences in nonparametric data. Individual data points representing biological replicates are plotted with the mean ± SEM, except for growth data, where, for clarity purposes, only the mean ± SD was plotted. Significance was determined at *p* ≤ 0.05.

## 3. Results

### 3.1. Iron Status

**Figure 1:**
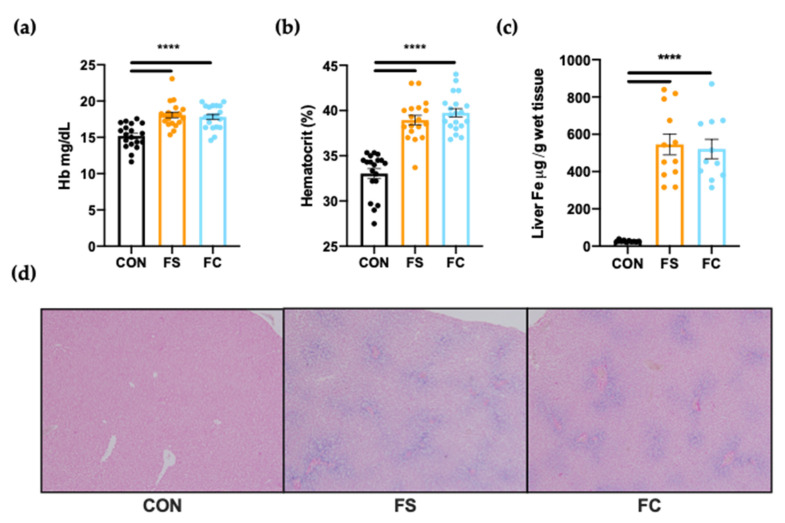
Iron status on postnatal day (PD) 15 following daily ferrous sulfate (FS), ferrous bis-glycinate chelate (FC), or vehicle control (CON) supplementation in rats from PD 2–14. (**a**) Hemoglobin and (**b**) hematocrit were measured from fresh whole blood (*n* = 20/group). (**c**) Liver iron concentrations were quantified by atomic absorption spectrometry (*n* = 12/group). Values are plotted as the means ± SEM. (**d**) Representative microscope images of liver sections stained with Perls’ Prussian blue for the detection of ferric iron deposits were captured using a 10× objective lens (*n* = 6/group). *p*-value summary: ****, *p* < 0.0001.

We provided daily ferrous sulfate (FS) or ferrous bis-glycinate chelate (FC) iron supplements to rat pups from postnatal day (PD) 2–14 to investigate outcomes of postnatal iron supplementation. Supplements were delivered based on 10 mg iron/kg body weight (BW), a dose we designed to represent the estimated routine iron intake of an infant fed exclusively iron-fortified infant formula (Table 1 and Equation (1)). We interrogated hemoglobin and hepatic iron pools to evaluate body iron stores at PD 15 following supplementation. Initially, we tested whether differences in liver iron, hemoglobin, and hematocrit may be due to sex. We did not detect any effects on these metrics due to sex, so this variable was dropped when testing for differences among iron supplementation groups. Hemoglobin and hematocrit were 15% higher in iron-supplemented pups (FS and FC) over CON (*p* < 0.0001; Figure 1a,b). Substantial liver iron loading was also observed in all iron-supplemented pups (Figure 1c,d). Liver iron concentration following FS or FC supplementation was around 100× CON liver iron levels (*p* < 0.0001; Figure 1a), and marked ferric iron deposition blue was clearly visible with Perls’ Prussian Blue iron staining in both FS and FC liver sections while nearly undetectable in CON livers (Figure 1d). No difference in hemoglobin (*p* = 0.87; Figure 1a), hematocrit (*p* = 0.27; Figure 1b), or liver iron concentration (*p* = 0.93; Figure 1c) was found between FS vs. FC groups (*p* = 0.93), suggesting that both iron forms elevated body iron levels similarly following daily supplementation.

### 3.2. Growth and Development

**Figure 2:**
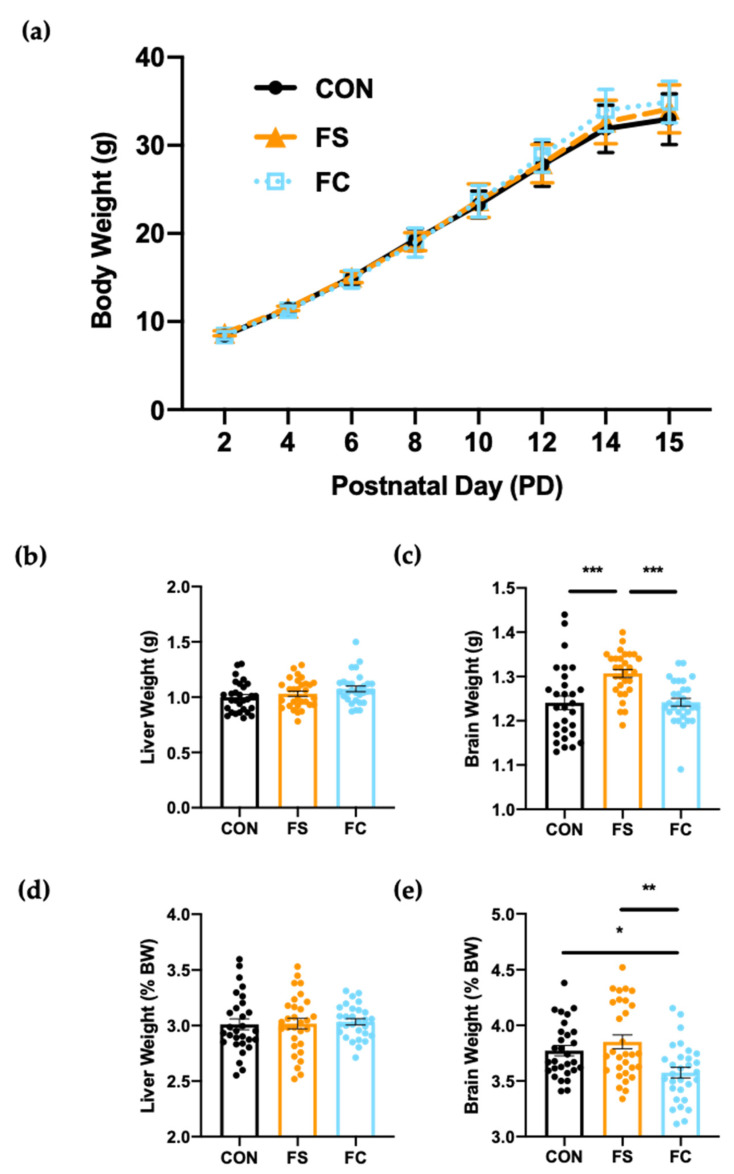
Weight gain and organ development with daily postnatal FS or FC supplementation in rats. (**a**) Pup body weights (*n* = 3 litters/group, 10 pups each litter) were recorded across the supplementation period from postnatal day (PD) 2–15; litter averages were analyzed as biological replicates and plotted as mean ± SD. Group and time effects were assessed by repeated-measures two-way ANOVA with Geisser–Greenhouse correction. (**b**) Liver and (**c**) brain weights were recorded at time of collection on PD 15 and normalized to body weight (**d**,**e**). Organ weight values are plotted as the means ± SEM. *p*-value summary: *, *p* < 0.05; **, *p* < 0.01; ***, *p* < 0.001.

Iron supplementation can delay growth when provided to iron-sufficient infants [12,13,14], and therefore we recorded BW every two days across this study to investigate the influence of supplementation on growth. Litter average BW increased steadily in all litters from postnatal day (PD) 2 to PD 15 (Figure 2a) and all individual pup weights fell within normal growth curve percentiles for Sprague Dawley rats (individual values not plotted for clarity). Litter averages were analyzed as biological replicates when testing for treatment effects on BW. A repeated-measures two-way ANOVA of litter average BW detected a significant effect of time (*p* < 0.0001) but not litter group (*p* = 0.18), suggesting that postnatal BW gain was not affected by iron.

Organ weights were measured on PD 15 at time of collection to detect organ toxicity effects [61]. No effect of sex on liver or brain weight was detected at this age. Treatment influenced brain weight, but results of pairwise comparisons were affected when raw brain weight values were normalized to BW. Mean FS brain weight (raw weight in g) was greater than in the FC and CON groups (*p* < 0.05; Figure 2c). However, mean FS brain weight (% BW) was not different from CON, and FC brain weight (% BW) was significantly lower than both FS (*p* < 0.01) and CON (*p* < 0.05; Figure 2e). With or without normalization, FS brains were significantly heavier than FC brains. Liver weight, in contrast, was not different between groups (*p* = 0.10; Figure 2b), and this remained true when values were normalized to body weight (*p* = 0.99; Figure 2d). Overall, brain development was affected by iron supplement form and this effect does not appear to be related to iron status, since iron status was similar between the FS and FC groups (Figure 1a–d).

### 3.3. Systemic Iron Homeostasis

**Figure 3:**
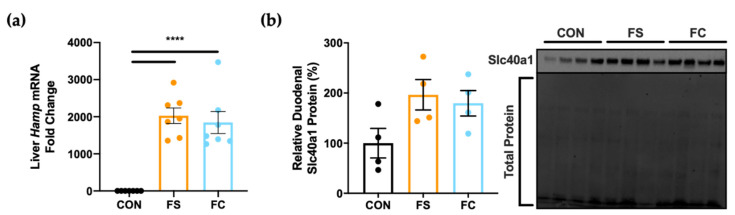
Changes in systemic iron regulation at PD 15. (**a**) Liver hepcidin (*Hamp)* mRNA expression was assessed by real-time PCR (*n* = 7–8/group). Values with the mean ± SEM are plotted as fold change relative to CON means. (**b**) Relative expression of the iron exporter protein, ferroportin (Slc40a1), was assessed in the proximal small intestine (*n* = 4/group). Adjusted Slc40a1 band density was normalized to total protein with the Stain-Free™ method, and values are plotted relative to mean CON expression (%) as the means ± SEM. *p*-value summary: ****, *p* < 0.0001.

When iron stores become elevated in healthy individuals, the liver releases the iron regulator hepcidin to prevent iron overload [62,63]. Hepcidin reduces dietary iron uptake by blocking activity of the iron exporter ferroportin in enterocytes [62,64] and inherited disruptions to this pathway result in hemochromatosis (i.e., iron overload) [65,66,67,68,69]. We assessed liver hepcidin (Hamp) and duodenal ferroportin (Slc40a1) expression in rats at PD 15 to observe systemic iron homeostasis following daily postnatal iron supplementation, and to test for differences between FS and FC. Hamp was increased by at least 1000-fold in FS and FC pups (*p* < 0.0001; Figure 3a), but no difference was found between iron groups. We did not observe a treatment effect on duodenal Slc40a1 expression (*p* = 0.09; Figure 3b), in support of findings suggesting that iron absorption is under-regulated in early life [25,28,30,31]. It appears that duodenal Slc40a1 trended toward increased expression with iron supplementation, but due to the small sample size (*n* = 4 per group), it is possible that our Slc40a1 analysis was underpowered to detect a significant change. Further, iron homeostasis outcomes of iron supplementation may not depend upon iron form.

### 3.4. Iron and Trace Minerals in the Central Nervous System

Next, we measured iron levels in the CNS at PD 15 to determine whether postnatal iron supplementation led to sustained brain iron loading, but in spite of increased overall iron status this was not the case. Indeed, no difference was found in whole brain iron concentrations among groups (*p* = 0.91), suggesting that, in contrast to the liver, the CNS may be protected from iron loading following postnatal supplementation at physiological doses.

We suspected that iron supplementation might reduce availability of other trace minerals in the CNS, as iron can disrupt the metabolism of other essential trace minerals through mineral-mineral interactions [15,20]. To test whether availability of these minerals was altered in the CNS following postnatal iron supplementation, zinc (Zn), copper (Cu), and manganese (Mn) concentrations were also quantified in whole brains. Congruent with brain iron results, brain zinc (*p* = 0.28), manganese (*p* = 0.84), and copper (*p* = 0.34) concentrations were unaffected by iron supplementation at this age.

### 3.5. Iron Regulation in the Hippocampus

**Figure 4:**
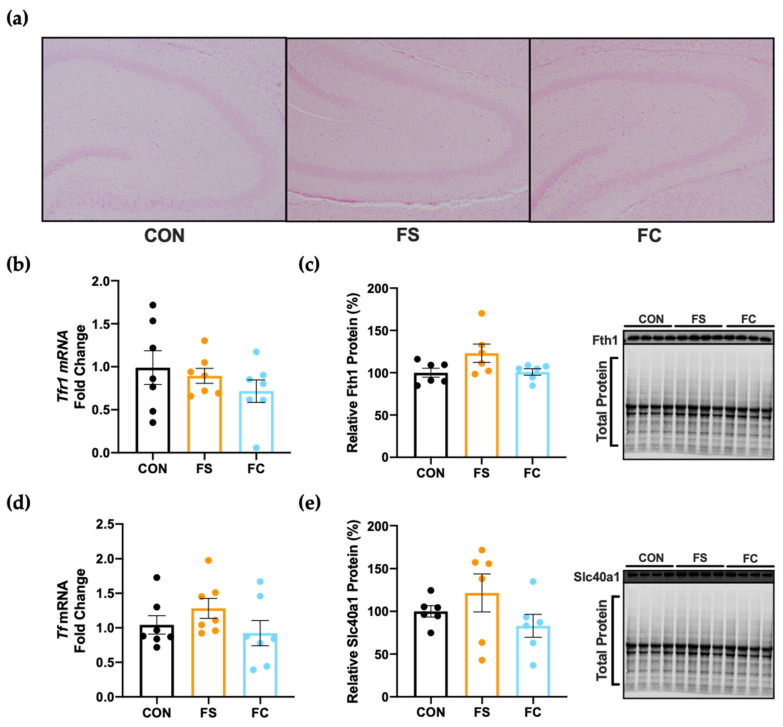
Hippocampal iron loading and regulation on PD 15. (**a**) Representative microscope images of hippocampal sections stained with Perls’ Prussian blue stain for ferric iron detection, captured with 10x objective lens (*n* = 5–6/group). (**b**) Transferrin receptor (*Tfr1*) and (**d**) transferrin (*Tf*) mRNA expression in hippocampal tissue, shown as fold change relative to CON (*n* = 7/group). (**c**) Hippocampal expression of ferritin heavy chain (Fth1) subunit, and (**e**) iron exporter protein ferroportin (Slc40a1) expression were normalized to total protein using the Stain-Free™ method and plotted relative (%) to CON expression (*n* = 6/group). Representative blots shown on the right with total protein. Values are shown as the means ± SEM.

Iron must be tightly regulated in the CNS to sustain basic cellular functions, neurotransmitter synthesis, and myelination. The hippocampus—a CNS region known for its central role in learning and memory—is considered highly sensitive to changes in iron availability during early development and aging. Hippocampal iron deficiency (ID) can permanently disrupt cognitive development, while hippocampal overload is a key component in Alzheimer’s Disease pathophysiology. We assessed iron loading and iron regulation in the hippocampus at PD 15 to observe whether the hippocampus had sustained iron loading following postnatal iron supplementation. Ferric iron deposits were undetectable in hippocampal sections (representative slides shown in Figure 4a), and no effect on hippocampal ferritin heavy chain protein (Fth1) expression was observed (*p* = 0.07; Figure 4c). This suggests iron loading did not occur in the hippocampus following iron supplementation, because iron is stored in ferritin and its components are upregulated in response to increased iron [70,71,72]. In addition to storing iron as Ft, the CNS can prevent iron overload during increased iron status by downregulating transferrin-bound iron uptake by transferrin receptor (Tfr1) or by increasing iron export via Slc40a1; transferrin is also upregulated in the CNS to quench free iron molecules during cellular iron overload or oxidative stress [54,73,74]. We found no differences in Tfr1 (*p* = 0.42; Figure 4b) or Tf (*p* = 0.27; Figure 4d) mRNA expression among groups, nor did we observe changes in Slc40a1 protein (*p* = 0.24; Figure 4e). Taken together, these data do not indicate sustained iron loading had occurred in the hippocampus following postnatal iron supplementation.

### 3.6. Oxidative Stress in the Hippocampus

**Figure 5:**
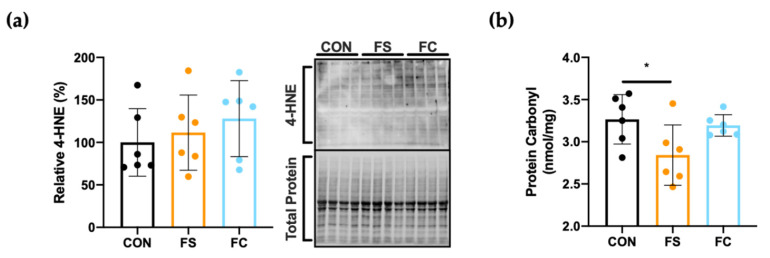
Hippocampal oxidative stress at PD 15. (**a**) To detect lipid peroxidation effects, 4-hydroxynonenal (4HNE) modified proteins were quantified by Western blot (*n* = 6/group), normalized to total protein using the Stain-Free™ method, and values with the mean ± SEM are plotted relative to mean CON expression (%). A representative blot with Stain-Free™ total protein blot is shown to the right. (**b**) Protein carbonyl content, a marker of protein oxidation, was quantified in hippocampal tissue lysates by enzyme-linked immunosorbent assay (ELISA; *n* = 6/group). Values are plotted as the means ± SEM. *p*-value summary: *, *p* < 0.05.

Iron induces oxidative damage in the CNS, including the hippocampus, and this may cause neurodegeneration [32], so we reasoned that postnatal iron supplementation might elevate oxidative stress in the hippocampus even in the absence of sustained iron loading effects, as this may occur through transient increases in CNS iron undetected by our study design. Hippocampal oxidative stress was quantified by measuring 4-hydroxynonenal (4HNE), a known product of lipid peroxidation [75]. The quantity of 4HNE modified proteins, assessed by Western blot, did not differ among groups (*p* = 0.54; Figure 5a); however, a slight effect on protein carbonyl content, a stable byproduct of protein oxidation, was observed [76]. Less oxidized protein was detected in the hippocampus of FS pups compared to the other groups, suggesting reduced hippocampal oxidative stress in this group (*p* = 0.04; Figure 5b).

### 3.7. Myelination in the Hippocampus

**Figure 6:**
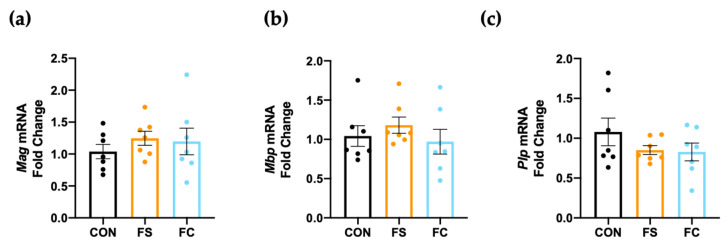
Hippocampal myelination gene expression at PD 15. Expression of major myelin components, (**a**) myelin-associated glycoprotein (*Mag*), (**b**) myelin basic protein (*Mbp*), and (**c**) myelin proteolipid protein (*Plp*) as assessed in hippocampal tissue by real-time PCR (*n* = 7/group). Values are plotted as fold change relative to CON as the means ± SEM.

Myelination occurs mainly during postnatal development in rats and synthesis of myelin peaks beginning PD 14 until PD 34 [77]. Iron accumulation causes oxidative stress and cell death in oligodendrocytes, which myelinate neurons in the CNS [78]. In piglets, iron supplementation reduced hippocampal myelination gene expression [38]. We sought to determine if hippocampal myelination was impacted by daily postnatal iron supplementation in rats, so we measured expression of several major myelin genes in the hippocampus, including Mag, Mbp, and Plp (Figure 6). Myelin associated glycoprotein (Mag) signals myelin and axonal formation, while myelin basic protein (Mbp) and proteolipid protein (Plp) play major structural roles in myelination [79]. We found no difference in expression of Mag, Mbp, or Plp mRNA in the hippocampus at PD 15, suggesting that myelination was not impacted by either iron supplement (*p =* 0.69; Figure 6).

## 4. Discussion

Ferrous sulfate (FS) supplementation and formula fortification prevent postnatal iron deficiency (ID) [4,6], but may be harmful to iron-replete infants [15,18,20]. Excess iron intake through high-iron formula or iron drops can lead to growth delays, and adverse cognitive and behavioral outcomes [9,10,11,19]. Infants may be especially susceptible to these adverse effects, because under-regulation of iron in early life permits excessive iron absorption and this may lead to iron loading in the developing central nervous system (CNS) [8,25,31]. Adverse neurodevelopment outcomes [23,24,25] and oxidative stress of the CNS [25,38] have been observed in animals supplemented with iron postnatally. Research on the effects of postnatal iron supplementation in healthy subjects is limited and existing animal studies have often not been designed to mimic routine iron administration. We developed a translationally-optimized iron supplementation experiment in rat pups (Table 1 and Equation (1)) to compare effects of ferrous bis-glycinate chelate (FC) or ferrous sulfate (FS) on development, systemic iron regulation, CNS trace mineral content, and hippocampus-specific markers of iron regulation, oxidative stress, and myelination.

First, we characterized iron status following supplementation with FS or FC. Hemoglobin, hematocrit, and liver iron content were all substantially increased in iron-supplemented pups at PD 15 (Figure 1). Liver iron concentration is more sensitive and specific to excess body iron loading than blood indices for iron status. Excess body iron is taken up by the liver for storage, and in turn, the liver controls body iron homeostasis to prevent overload. Before being assigned to treatment groups on PD 2, litters were culled to age-matched litters of 10, a normal litter size for Sprague Dawley rats. Therefore, it can be assumed that CON pups received sufficient dietary iron via milk feeding and should not have required additional iron. Yet, we observed large effects on hemoglobin, hematocrit, and liver iron content when pups were supplemented with iron, and this is probably due to under-regulation of iron absorption (Figure 3). When liver iron increases, the liver makes hepcidin, the iron systemic iron regulator that downregulates intestinal iron absorption by blocking the iron exporter, ferroportin (Slc40a1) [20,21,22]. Infant iron absorption was previously reported to be unaffected by dose, mode of delivery, or infant iron status [27,80], and in previous experiments in rodents [29,30,31] and piglets [25] intestinal ferroportin was hypo-responsive to hepcidin following iron supplementation. One study investigating this early life phenomenon in rats concluded that hypo-responsiveness of ferroportin protein to hepcidin during suckling may be explained by elevated iron-regulatory element (IRE+) Slc40a1 transcripts, which allow for upregulation of Slc40a1 in response to elevated enterocyte iron levels [29]. That study demonstrated that weanling and adult rats mainly express an Slc40a1 transcript variant lacking IRE (IRE−) in the duodenum. Expression of IRE− Slc40a1 in weanling and adult rats allows enterocytes to avoid translational regulation by iron regulatory proteins (IRPs); enterocyte Slc40a1 protein is primarily controlled by hepcidin after weaning. However, in pre-weanling pups expressing higher levels of IRE+ Slc40a1 transcripts, translation of Slc40a1 is upregulated in response to iron; Slc40a1 remains elevated even in the presence of elevated hepcidin levels. The authors concluded that elevated IRE+ Slc40a1 during suckling may help to maximize the supply of iron during a critical period of increased iron demands [29]. In our study, liver iron concentrations in FS and FC pups were 100x control (CON) levels (Figure 1c), and liver hepcidin expression was 1000-fold CON expression, but we found no change in intestinal ferroportin protein (Figure 3). Indeed, there was a trend toward increased ferroportin expression (Figure 3b); however, our duodenal Western blot analysis may have been underpowered to detect a significant increase. Therefore, our results are consistent with previous findings that infants receiving iron through iron supplements or iron-fortified formula absorb iron unmitigatedly and may be at increased risk for iron overload. Both FS and FC supplementation comparably increased iron levels, hemoglobin, hematocrit (Figure 1), and similar liver *Hamp* expression and duodenal ferroportin protein expression was detected between FS and FC groups (Figure 3). Indeed, neither iron status nor iron homeostasis outcomes were affected by iron form; both forms elevated body iron stores to levels far beyond that of CON pups.

Iron deficiency and iron toxicity are both harmful, and both inhibit growth and proliferation of cells [81]. A limited number of studies have investigated whether postnatal iron supplementation benefits long-term growth and development [18,82,83,84]. Iron supplementation of iron-sufficient infants might delay growth but this is not consistent [9,12,14,82,83,85]. We observed no effect of iron supplementation on litter weight gain (Figure 2a), suggesting that neither FS nor FC iron affects short-term weight gain in early life when provided at routine levels. Similar findings have been reported in previous animals studies, which have used both lower and higher daily doses of FS: in pre-weanling pigs—where the same daily dose of FS was used (10 mg iron/kg BW) from PD2–21—weight gain was not affected, nor was weight gain affected with 50 mg iron/kg BW [25], and BW was not affected in pre-weaning rats following supplementation with either 30 or 150 µg iron per day [30]. In these studies, increasing the dose of iron increased iron status but did not change growth. Thus, our results are consistent with previous experiments in animals. We also analyzed liver and brain weights following iron supplementation in rats, because organ weight is often measured to detect neonatal toxicity in rodent models [61]. Liver size typically decreases with exposure to environmental toxins [86]. We observed no difference in liver weight following iron supplementation, indicating an absence of toxic effects in the liver (Figure 2b,d). Nevertheless, brain weight was affected depending on iron form (Figure 2c,e). Data are shown as brain weight and brain % BW because current research has not determined which is more meaningful in terms of postnatal neurodevelopment [61]. In both analyses (Figure 2c,e), FS brains were heavier than FC brains. Therefore, we conclude that brain weight effects following postnatal iron supplementation are dependent upon the form of iron. Additional studies with more specific indicators of neurodevelopment are needed to determine whether functional differences may arise related to brain weight or iron source.

Previous studies have observed iron loading in the CNS following iron supplementation [25,30] and this may also alter availability of other trace metals through iron–mineral interactions [15,20]. We reasoned that differences in brain size between the iron forms might be explained by differences in iron loading or trace metal availability between FS and FC groups, but neither iron, zinc, copper, nor manganese levels were different between these groups in our study. Regarding the negative cognitive and behavioral outcomes that were observed in infants given iron-fortified formula, these results suggest dietary iron intake from iron-fortified formula is unlikely to have caused sustained brain iron loading or disruptions to zinc, copper, or manganese availability in the CNS. Furthermore, these findings do not support the hypothesis that long-term cognitive outcomes of postnatal iron supplementation are due to direct effects of iron loading or iron–mineral interactions in the CNS. Neither does it appear likely that sustained iron loading nor changes in iron regulation had occurred specifically in the hippocampus, as we had suspected it would (Figure 4). Postnatal CNS iron loading might happen transiently, or after exceptionally high oral doses are used as previously reported [25,30]. Considering that brain trace minerals were not altered and considering that overall iron status was similar between FS and FC groups, it is unclear how FS brains became heavier than FC brains. These results provide novel evidence that iron form might influence neurodevelopment outcomes of iron supplementation.

Iron loading causes oxidative stress in the brain and this appears to be a central mechanism in neurodegenerative pathologies [32,33]. Oxidative stress has also been observed in the CNS following neonatal iron exposure [87]. Iron loading initiates pro-oxidative reactions in cells and this can be toxic to the CNS [78,88,89]. Recently in piglets, iron supplementation at 50 mg iron/kg BW as FS from PD 2 to PD 21 increased hippocampal lipid peroxidation compared to 10 mg iron/kg and control groups, but this was not statistically significant [25]. In the present experiment, we used 10 mg iron/kg BW. No change in hippocampal lipid peroxidation was observed, and only borderline less hippocampal protein oxidation was seen in the FS group, suggesting that neither iron treatment induced hippocampal oxidative stress (Figure 5). Protein oxidation was not different among the iron groups in the hippocampus, so we further conclude that differences in brain size cannot be explained by differences in oxidative stress outcomes between iron forms. Congruent to both these and the CNS mineral loading results, we also did not detect changes in myelination gene expression (Figure 6).

There are inherent limitations to extending the findings of this study to all healthy, iron-sufficient infants in spite of our optimization efforts. We believe that 10 mg iron/kg BW is representative of the iron intake of iron-fortified formula-fed infants, but dietary iron intake may vary widely in healthy infants. It is possible that many infants may be exposed to significantly more iron (e.g., preterm infants) or less iron (mixed-fed infants) than the average formula-fed infant. It is likely that significantly increasing or decreasing the dose used in our study would lead to different iron status and development outcomes. Future studies should define the dose–response relationship between postnatal iron intake, iron status, growth, and neurodevelopment at this stage of life.

In conclusion, specific development effects of postnatal iron supplementation at routine levels may not be clearly related to iron status effects and instead dependent upon indirect mechanisms related to iron form. The long-term functional consequences of these effects remain to be elucidated. The differential effects on brain growth between FS- and FC-supplemented pups provides evidence that iron impacts postnatal development in a form dependent manner. Additional studies in this area are warranted to optimize dose, timing, and form of iron for infants such that any negative health outcomes are identified and prevented without compromising risk for iron deficiency.

## Figures and Tables

**Table 1:** The daily rat pup iron supplementation dose—10 mg elemental iron per kg body weight (BW)—was determined by estimating Adjusted Daily Iron Intakes of human milk (HM), iron-fortified formula (IF), and rat milk (RM) diets; these values were then used to calculate a daily supplemental iron intake for nursing rat pups that is proportional to the Adjusted Daily Iron Intake in infants fed exclusively IF relative to HM-fed infants (Equation (1)). Recommended iron intakes and experimental iron overload doses for adult rats are included for comparison and reference.

Species	Diet	Diet Intake	Dietary Iron (ppm)	Daily Iron Intake ^1^ (mg/kg BW)	Iron Absorption (%)	Adjusted Daily IronIntake ^2^ (mg/kg BW)
Human	Human milk (HM)	600–800 mL [6]	0.35 [44]	0.04–0.07	50 [27]	0.02–0.035
Human	Iron-Fortified Formula (IF)	600–800 mL [6]	12 [7]	1.4	10 [28]	0.14
Rat (pup)	Rat Milk (RM)	4–10 mL [45]	5 [46]	1.6–2.0	100 [30]	1.6–2.0
Rat (adult)	Standard Chow	25–30 g [47]	35–250 [48]	3.5–21	100 [49]	3.5–21
Rat (adult)	Iron Overload	25–30 g [47]	8000–10,000 [50,51]	800–2500	NA	NA

^1^ Estimations based on 5–7 kg infant; 10–30 g rat pup; 250–300 g adult rat. BW = Body Weight. ^2^ Values have been adjusted for iron absorption.

**Table 2:** Real-time PCR primer sequences.

Gene	Primer Sequence	Reference
*Actb*	F: GAAATCGTGCGTGACATTAAAGAG R: GCGGCAGTGGCCATCTC	[53]
*Hamp*	F: GCTGCCTGTCTCCTGCTTCT R: CTGCAGAGCCGTAGTCTGTCTCGTC	[29]
*Tf*	F: GCATCAGACTCCAGCATCAA R: CAGGACAGTCTGGTGCTTCA	[54]
*TfR1*	F: GAGTTCACTGACATCATCAA R: GCAATCCAGATGACTGAGAT	[53]
*Mag*	F: TGTGTAGCTGAGAAGGAGTATGG R: ACAGTGCGATTCCAGAAGGATTAT	[55]
*Mbp*	F: CTCTGGCAAGGACTCACACAC R: TCTGCTGAGGGACAGGCCTCTC	[56]
*Plp*	F: GTGTTCTCCCATGGAATGCT R: TGAAGGTGAGCAGGGAAACT	[57]

## Data Availability

The data presented in this study are not publicly available. The data are available on request from the corresponding author.
